# Preferential synthesis of (6,4) single-walled carbon nanotubes by controlling oxidation degree of Co catalyst

**DOI:** 10.1038/s41598-017-11712-0

**Published:** 2017-09-11

**Authors:** Bin Xu, Toshiro Kaneko, Yasushi Shibuta, Toshiaki Kato

**Affiliations:** 1 Department of Electronic Engineering, Tohoku University, Aoba 6-6-05, Aramaki, Aoba-ku, Sendai 980-8579 Japan; 2 Department of Materials Engineering, The University of Tokyo, 7-3-1, Hongo, Bunkyo-ku, Tokyo 113-8656 Japan

## Abstract

Chirality-selective synthesis of single-walled carbon nanotubes (SWNTs) has been a research goal for the last two decades and is still challenging due to the difficulty in controlling the atomic structure in the one-dimensional material. Here, we develop an optimized approach for controlling the chirality of species by tuning the oxidation degree of Co catalyst. Predominant synthesis of (6,4) SWNTs is realized for the first time. The detailed mechanism is investigated through a systematic experimental study combined with first-principles calculations, revealing that the independent control of tube diameter and chiral angle achieved by changing the binding energy between SWNTs (cap and tube edge) and catalyst causes a drastic transition of chirality of SWNTs from (6,5) to (6,4). Since our approach of independently controlling the diameter and chiral angle can be applied to other chirality species, our results can be useful in achieving the on-demand synthesis of specific-chirality SWNTs.

## Introduction

**Figure 1:**
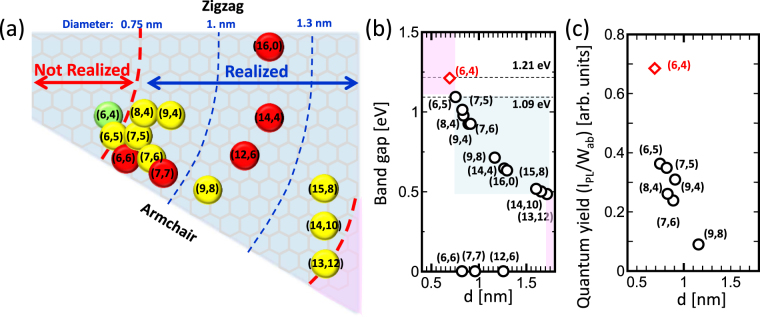
Summary of the preferential synthesis of SWNTs. (**a**) Map of chirality species obtained for relatively narrow chirality-distributed growth (yellow) and high purity synthesis (red) of SWNTs. (**b**,**c**) Plot of (**b**) band gap and (**c**) quantum yield vs. d for the SWNTs chiralities in which preferential growth was realized.

Owing to the excellent electrical and optical properties reported by many researchers^1, 2^, single-walled carbon nanotubes (SWNTs) are expected to be utilized in a wide range of applications, e.g., in thin film transistors, photodetectors in THz range, and chemical sensors^3–5^. Even though many studies have been performed on them over the last two decades, the selective growth of SWNTs with a specific chirality, which determines the physical and chemical properties of SWNTs such as band gap, carrier mobility, quantum yield, and chemical reactivity, is still challenging. Gradual progress has been made for achieving chirality-controlled synthesis of SWNTs. Figure 1 shows the chirality map (Fig. 1a) and band gap (Fig. 1b) of 14 different chiral species, where a preferential synthesis has been reported thus far. The approaches employed for the preferential synthesis of SWNTs with a specific chirality can be divided into two groups, which is (1) catalyzed growth and (2) templated growth (Table S1). Catalyzed growth is a kind of chemical vapor deposition (CVD) synthesis, which has been employed by controlling various parameters such as the catalysts and gas phase reaction^6–17^. Templated growth can be further categorized into two groups namely (2-1) cloning growth and (2-2) growth from chemically derived cap. The cloning growth uses a mother crystal containing chiral-selective SWNTs obtained by a post-separation process^18–20^, realizing high purity synthesis of (6,5), (7,6), and (7,7) SWNTs^19^. The pre-designed specific-cap structures are also used as templates for CVD growth for obtaining (6,6) SWNTs with a very high purity^21^. Although several promising results have been obtained using the templated growth, a low growth yield is usually achieved. Hence, further progress should be made for practical applications. A higher yield of growth can be achieved with the catalyzed growth as compared to the templated growth. Due to the recent progress in the catalyzed growth, chirality control of SWNTs has been significantly improved, giving rise to narrow-chirality distributed SWNTs (Fig. 1). The main approach of catalyzed growth can be divided into three groups, which is (1-1) using various kinds of catalysts, (1-2) gas phase control during the growth, and (1-3) crystal phase control of catalyst. For (1-1) using various kinds of catalysts, (6,5), (7,5), and (7,6) SWNTs can be preferentially grown using bi-metal catalysts of Co and Mo (CoMoCAT)^6^. Addition of a small amount of impurities such as Mn and Cr to Co can result in the formation of (6,5) dominant SWNTs^7, 8^. Au catalyzed growth with hydrogen-assisted plasma CVD can realize the preferential synthesis of (6,5) SWNTs^10^. On mixing S with Co, (9,8) SWNTs can be obtained^12^. Relatively large diameter (d) SWNTs with (14,10) and (15,8) chiralities can be dominantly grown with Rh catalyst^13^. The second one, (1-2) gas phase control, can also effect on reducing chirality distribution. For example, by mixing NH_3_ gas during CVD, enhanced growth of large diameter SWNTs with (13,12) SWNTs as the dominant chirality has been achieved^14^. The (7,6) and (8,4) SWNTs can also be preferentially grown by precisely controlling the growth time during the incubation period in plasma CVD^11^. The third one, (1-3) crystal phase control, becomes one of the main approaches for chirality selective synthesis at the current state. Highly-crystalline catalyst (Co_x_Mg_1-x_O) can obtain (6,5), (7,6), and (9,4) SWNTs^9^. W-based bi-metallic alloy catalysts maintain their crystalline structures during nucleation, resulting in the formation of (12,6), (14,4), and (16,0) SWNTs with a high selectivity^15–17^. Although the use of a specific crystal phase of catalysts is promising to achieve chirality-selective nucleation, the number of chiralities match with the specific-crystal phase should be limited, and most of these chiralities correspond to relatively larger d of >1 nm (Fig. 1a). Therefore, it is still challenging to develop a method for synthesizing SWNTs with a specific chirality, which can be used for producing various chiral species, especially in the case of species for which a preferential synthesis has not yet been realized. For example, the growth of SWNTs with a band gap higher than that of (6,5) (current maximum) or lower than that of (13,12) SWNTs (current minimum) is highly required for semiconductor applications (Fig. 1b).

Here, we report the preferential growth of (6,4) SWNTs with high purity (∼57%) achieved by precisely controlling the oxidation degree of Co, where the basic concept itself is different from previous catalyzed growth controlling (1-1) catalyst type, (1-2) gas phase reaction, and (1-3) crystal phase of catalyst. (6,4) SWNTs have the smallest d among SWNTs formed with preferential synthesis as reported thus far (Fig. 1a). Since the band gap of semiconductor SWNTs is inversely proportional to the d, the preferential synthesis of (6,4) SWNTs has applications in SWNTs requiring a wider band gap (Fig. 1b)^22^. The high quantum yield (calculated from theoretically predicted value of photoluminescence (PL) intensity (I_PL_) and absorption efficiency (W_ab_) (I_PL_/W_ab_)) of (6,4) SWNTs^23^ (1.89 times higher than that of (6,5), which is the highest value achieved for the preferential synthesis of specific-chirality SWNTs) can also contribute in developing future high-performance optoelectrical devices using chirality-controlled SWNTs (Fig. 1c). The detailed mechanism for the chirality-selective synthesis is investigated. Systematic experimental studies and theoretical calculations based on first-principles calculations revealed that the diameter and chiral angle of SWNTs can be independently tuned by changing two of binding energies, namely, between the initial cap structure and catalyst surface (E_BC-M_) as well as between the nanotube edge and catalyst surface (E_BE-M_), resulting in a drastic transition from (6,5) to (6,4) SWNTs. Our result is very important in terms of not only achieving the dominant growth of (6,4) SWNTs but also providing a new strategy for the chirality-selective synthesis of SWNTs, thereby contributing to the on-demand for the synthesis of specific-chirality SWNTs, which is regarded as an ultimate goal.

SWNTs were synthesized using the diffusion plasma CVD method developed by our group (details are given in Methods)^9, 10^. Zeolite-supported Co and Co-Mo bi-metal (CoMo) catalyst were used for the SWNT growth. The chirality of the SWNTs was estimated using photoluminescence-excitation (PLE) mapping and ultraviolet-visible-near inferred (UV-Vis-NIR) spectroscopy. The catalyst morphology was evaluated using transmission electron microscopy (TEM). Surface states of the catalysts were examined using X-ray photoelectron spectroscopy (XPS), energy dispersive X-ray spectrometry (EDX), X-ray absorption fine structure (XAFS), and extended XAFS (EXAFS) (see Methods section for detailed information).

## Results

Since the key strategy used in this study for achieving chirality-controlled synthesis is to control the catalyst surface state that is related to chemical reactivity, we tried various gas-phase pretreatment methods prior to the synthesis of SWNTs by varying gas species, treatment temperature, and process time. To understand the effect of gas species used in pretreatment on chirality selection, a quadrupole mass (Q-mass) analyzer was used to monitor the gas species present during the whole process in real time. Note that the gas species used during the pretreatment stages might vary as gases could come out from the chamber wall or sample holder. Hence, real-time monitoring with the Q-mass analyzer is necessary for precisely tuning the gas species used during pretreatment. By performing the experiment over 100 times, it is revealed that the chirality distribution of the as-grown SWNTs is sensitive to the gas species used in the pretreatment stage. The purity of (6,4) SWNTs is sensitive to the presence of H_2_O, H_2_, and N_2_ used in the pretreatment process (Fig. S1).

**Figure 2:**
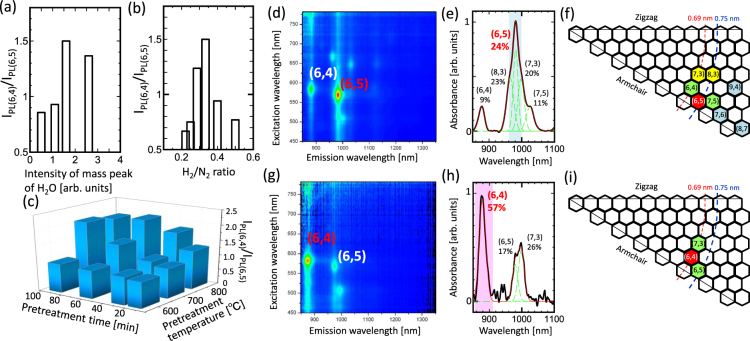
Chirality distribution of SWNTs synthesized using catalyst with and without undergoing pretreatment. (**a**–**c**). I_PL(6,4)_/I_PL(6,5)_ as a function of (**a**) H_2_O concentration, (**b**) H_2_/N_2_ ratio, as well as (**c**) temperature and time of pretreatment. (**d**,**g**) PLE maps, (**e**,**h**) UV-Vis-NIR spectra, and (**f**,**i**) chirality maps of SWNTs growth achieved (**d**–**f**) without and (**g**–**i**) with catalyst pretreatment. The colors shown in (**f**) and (**i**) indicate the concentration of different chiralities decreasing in the order of red, yellow, green, and blue.

To verify the detailed effects of gas species, the amount of each gas (H_2_O, H_2_, and N_2_) used for pretreatment was precisely controlled. Other conditions such as the parameters employed in plasma CVD, the catalyst composition, pretreatment temperature (800 °C), and pretreatment time (30 min) were unchanged in this experiment. On introducing a small amount H_2_O during the pretreatment stage with H_2_ and N_2_ mixture gas, the photoluminescence (PL) intensity ratio of the (6,4) SWNTs to (6,5) SWNTs (I_PL(6,4)_/I_PL(6,5)_) dramatically increases (Fig. 2a). H_2_/N_2_ ratio was then changed at a fixed H_2_O supply, and we obtained the highest I_PL(6,4)_/I_PL(6,5)_ with a particular H_2_/N_2_ ratio (Fig. 2b). From these results, we conclude that the selectivity of (6,4) and (6,5) SWNTs can be controlled by varying the gases employed during the pretreatment process. For further increasing the purity of (6,4) SWNTs, the pretreatment temperature and time were optimized using a fixed ratio of gases (H_2_O, H_2_, and N_2_). The purity of (6,4) SWNTs can be largely enhanced at higher pretreatment temperatures (over 700 °C) and longer pretreatment times (>60 min) as shown in Fig. 2c.

Figure 2d–i show the typical PLE map, UV-Vis-NIR spectra, and chirality map of the SWNTs grown with non-pretreated catalyst and catalyst pretreated under the best conditions. In the case of SWNTs grown using a non-pretreated catalyst, the chirality distribution is relatively narrow with (6,5) SWNTs as the dominant chirality (Fig. 2d–f), which could be attributed to the use of zeolite supported CoMoCAT and the low growth temperature used in plasma CVD. In contrast, the most dominant-chirality species changed from (6,5) to (6,4) on using the pretreated catalyst (Fig. 2g–i) with the purity of (6,4) SWNTs reaching up to 57% (Fig. 2h). This is the first time result that (6,4) SWNTs with a high concentration could be grown. It should be also mentioned that our process has very high reproducibility, which can be caused by precise vacuum control with *in-situ* monitoring with Q-mass analyzer as mentioned above (Fig. S2).

To elucidate the growth mechanism of the preferential growth of (6,4) SWNTs, the variation in the catalyst structures and surface states were investigated with and without employing pretreatment. The pretreatment parameters were set as the one obtaining the high purity of (6,4) SWNTs, which is H_2_ = 1 sccm, N_2_ = 3 sccm, H_2_O = 2 × 10^−11^ counts (measured by Q-mass), pretreatment time = 30 min, and pretreatment temperature = 800 °C. For simplification purposes, we used Co as the catalyst instead of the CoMo bi-metal catalyst hereafter. Similar enhancement in the (6,4) SWNT growth was observed on using pretreated Co as the catalyst (Fig. S3). It is also confirmed that SWNTs cannot be grown using only Mo as the catalyst. This result is in good accordance with the previously reported studies indicating that Mo serve as a support for the Co catalyst rather than acting as the catalyst for the SWNT growth (Fig. S3)^24^.

**Figure 3:**
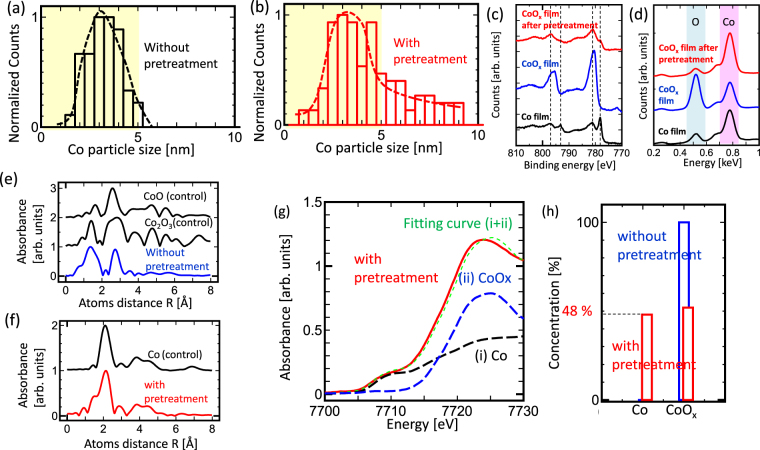
Analysis of the catalyst surface state. (**a**,**b**) Histogram of particle size obtained (**a**) without and (**b**) with catalyst pretreatment measured by TEM. (**c**,**d**) (**c**) XPS and (**d**) EDX spectra of Co film (black), oxidized-Co film (blue), oxidized-Co film obtained after pretreatment (red). (**e**,**f**) FT-EXAFS spectra of zeolite supported Co (**e**) without and (**f**) with pretreatment. Control spectra obtained from reference samples are shown in (**e**) CoO and Co_2_O_3_ and (**f**) Co. (**g**) XAFS spectra of zeolite-supported Co with pretreatment (red) and the fitted curve for (i) Co component (black dash), (ii) CoO_x_ component (blue dash), sum of (i + ii) (green dash), and reproduction of the experimental data (red). The curve of CoO_x_ component is same as that of non-treated zeolite supported Co. (**h**) Concentration of Co and CoO_x_ in zeolite supported Co without and with pretreatment as estimated by fitting the XAFS curve (**g**).

Since it is well known that the catalyst size is sensitive to the SWNT structures, the variation in the catalyst size was examined using TEM. After carrying out the pretreatment process, the Co nanoparticle size slightly increases even in the presence of zeolite support, which can prevent the aggregation of metal nanoparticles. However, the size distributions of the nanoparticles of diameters below 5 nm, which are assumed to affect the growth of SWNTs, remain almost unchanged (Fig. 3a and b). This indicates that the preferential growth of (6,4) SWNTs should be difficult to achieve by only changing the catalyst size. The selectivity of (6,4) and (6,5) SWNTs is hardly influenced by the catalyst size because the diameter difference between (6,4) and (6,5) SWNTs is as low as 0.065 nm. The effect of interaction between the supported material and CoMoCAT can be modified by employing pretreatment^24^. The difference in interaction between Mo and supported material causes changes in the morphology of Co nanoparticles present on the Mo under layer, resulting in different chirality distributions^24^. However, the enhancement in the growth of (6,4) SWNTs by pretreatment can be achieved even without using Mo (Fig. S3), indicating that the difference in interaction between Mo and supported material caused by pretreatment is not the critical factor determining the preferential growth of (6,4) SWNTs in our experiments.

Since the drastic change of chirality species from (6,5) SWNTs to (6,4) SWNTs can not be simply explained by the change of catalyst size distribution, the surface state of the catalyst was systematically analyzed as a next possible candidate causing the chirality transition. To understand the effect of pretreatment on the catalyst surface state, thin pure-Co film (~100 nm) and oxidized-Co film (~100 nm) were used instead of zeolite-supported Co nanoparticles for simplification. The pure-Co film was prepared by employing vacuum evaporation of Co on a SiO_2_ substrate. The oxidized-Co film was prepared by treating the pure-Co film with O_2_ plasma. A change in peak position can be clearly observed in the XPS spectra of the pure-Co film and oxidized-Co film (Fig. 3c). When the oxidized-Co film was pretreated under the best conditions needed for the (6,4) SWNT growth, an XPS peak similar to that of the pure-Co film was observed, indicating that the Co film was reduced during pretreatment (Fig. 3c). The samples were analyzed using EDX. A clear reduction in oxygen concentration is obtained after pretreatment (Fig. 3d). These results show that the reduction of oxidized Co should be occurring dominantly during the catalyst pretreatment process. Note that nitrogen could not be detected by EDX, indicating that the nitride reaction of Co does not significantly happen during the catalyst pretreatment process (Fig. S4).

To estimate the quantitative degree of reduction occurring on the real catalyst used for the SWNT growth, XAFS and EXAFS measurements of the zeolite-supported Co nanoparticles were carried out (Fig. 3e–h). The Fourier-transformed spectrum in the higher-energy side of XAFS spectra (FT-XAFS) reveals the atomic structures in detail. The non-pretreated zeolite-supported Co catalysts consists of Co monoxide and Co (III) oxide as the dominant species (Fig. 3e), whereas the zeolite-supported Co catalyst pretreated under optimized conditions is dominated by pure Co (Fig. 3f), which is in consistent with the XPS and EDX results. The accurate percentage of Co over oxidized Co can be obtained by analyzing the near absorption edge of XAFS spectra. In the case of untreated Co catalyst, pure Co could not be detected as it was below the detection limit and Co is found to exist mostly in the oxidized phase. However, the pure Co content increases to as high as 48% after performing pretreatment (Fig. 3g and h). Thus, the oxidized zeolite-supported Co catalyst undergoes partial reduction (up to 48%) with pretreatment.

It is well known that the reduction of oxidized metals can be easily realized by using H_2_ gas. However, the clear dependence of I_PL(6,4)_/I_PL(6,5)_ on H_2_/N_2_ ratio during pretreatment can be obtained (Fig. 2b). Hence, it is important to identify the role of each gas (H_2_, H_2_O, and N_2_) used in the pretreatment process. No reduction of the Co film can be achieved on performing pretreatment without H_2_ (H_2_O and N_2_ only), indicating that H_2_ plays a crucial role in the reduction process as reported earlier (Fig. S5)^25^. Inhomogeneous reduction occurs when pretreatment is carried out without H_2_O (H_2_ and N_2_ only), revealing that H_2_O improves the uniformity of reduction in the whole sample (Fig. S6) (the detailed mechanism for the uniformity improvement achieved with H_2_O is out of the scope in this study). The extent of oxidation can be varied by adding N_2_ to H_2_ during the pretreatment (Fig. S5). Thus, N_2_ acts as a dilution gas for H_2_, thereby controlling the oxidation degree on the Co surface. Similar enhancement in the (6,4) SWNTs growth can be obtained on using He instead of N_2_, confirming the role of N_2_ gas used during pretreatment (Fig. S7). Thus, it is not pure Co, but oxidized Co with a certain degree is assumed to play an important role in obtaining (6,4) rich SWNTs.

## Discussion

**Figure 4:**
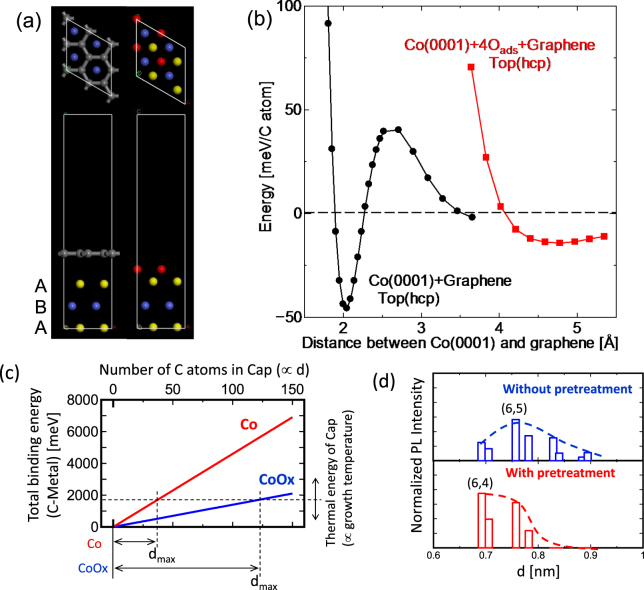
Experimental and theoretical investigations of the effect of catalyst pretreatment on SWNTs growth: Cap nucleation state. (**a**) Atomistic configuration of graphene and Co(0001) surface with and without oxygen atoms interacted by top(hcp) orientation. Blue and yellow spheres represent cobalt atoms in A and B layers, respectively, and gray and red ones represent carbon and oxygen atoms. Graphene in the system with oxygen atoms is not shown for the clarity of the image. (**b**) Binding energy per carbon atom between graphene and Co(0001) surface with and without oxygen atoms for top(hcp) orientation as a function of interlayer distance. (**c**) Total binding energy per each cap as a function of total number of carbon atoms in each cap (∝ d) for Co (red) and CoO_x_ (blue) catalyst. (**d**) Experimental data of normalized PL intensity as a function of d of SWNTs grown from zeolite-supported Co without (blue) and with (red) pretreatment.

To understand the reason for the drastic changes in chirality from (6,5) to (6,4) SWNTs achieved on tuning the oxidation degree of Co catalyst during pretreatment, the growth model is investigated by performing a systematic experimental study combined with first-principles theoretical calculations. Initially, we focus on the cap nucleation stage. Based on previous studies, the growth dynamics of SWNTs can be considered as follows^26^: A fullerene like semi-sphere cap structure is generated on the catalyst surface. The cap structure is then lifted off from the catalyst surface. Finally, the tube structure starts to grow in the axis direction. To lift off the cap structure from the catalyst surface, energy higher than the binding energy of the interlayer interaction between catalyst and cap structure is required^26^. The binding energy between the cap and catalyst is assumed to be sensitive to the oxidation degree of the catalyst. The binding energy between the cap and Co is obtained from first-principles calculations. Since the initial cap structure can be assumed as equivalent to that of graphene, we calculated the binding energy between graphene and Co crystal with and without the presence of oxygen atoms on the cluster surface (Figs S8, S9). The binding energy between graphene and oxidized Co is ∼14 meV/C, while a significant increase in the binding energy can be obtained with pure Co (∼46 meV/C) (Fig. 4a,b). To initiate the growth of SWNTs, the cap structure should be lifted off from the surface of the catalyst. Therefore, the energy required for lifting off the cap structure increases with pretreatment because of reduction of Co. The total binding energy required for the cap lift off can be obtained by adding the binding energy between each carbon atom in the cap and the catalyst surface (E_BC-M_). Larger-diameter caps contain greater number of carbon atoms (C_cap_) ((6,4):C_cap_ ∼ 30, (6,5):C_cap_ ∼ 43)^27, 28^, thereby requiring a higher energy for lifting off the large diameter SWNTs than that for the smaller ones. Thus, the upper limit of diameter for SWNTs grown under the same growth temperature should be down shifted by introducing the pretreatment due to the increase of E_BC-M_ (Fig. 4c). This is in good agreement with the experimental results, where the diameter distribution of SWNTs is slightly down-shifted after pretreatment due to the increase and decrease of small ((6,4)) and large diameter SWNTs ((8,3) and (7,5)), respectively (Fig. 4d).

We explained the effect of catalyst pretreatment on the preferential growth of smaller diameter SWNTs with an increase of E_BC-M_. However, other chirality species such as (5,5), (7,3), and (8,2) exhibit a similar diameter as that of (6,4). Thus, the transition from (6,5) to (6,4) SWNTs obtained in this study cannot be explained by only considering the diameter shift of SWNTs.

**Figure 5:**
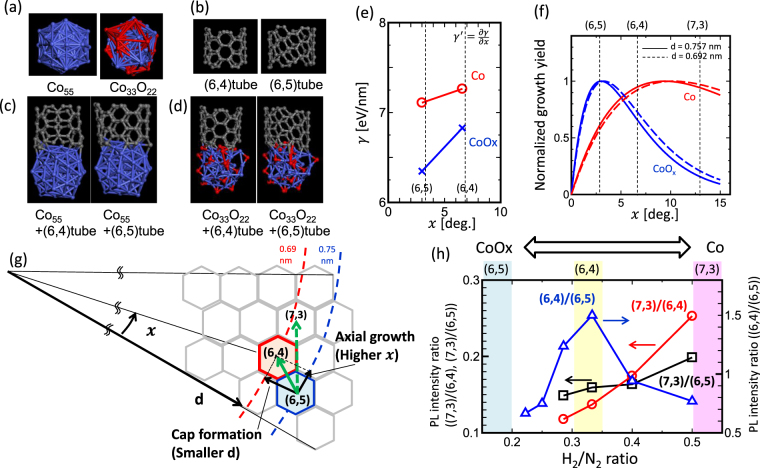
Experimental and theoretical investigations of the effect of catalyst pretreatment on SWNTs growth: Axial growth state. (**a**–**d**) Optimized structures from the first-principles calculations for (**a**) Co_55_ and Co_33_O_22_ clusters as well as (**b**) (6,4) and (6,5) SWNTs. (**c**,**d**) Binding structure between (**c**) Co_55_ and SWNTs and (**d**) Co_33_O_22_ and SWNTs. (**e**) The γ –*x* plot of Co and CoO_x_ obtained by first-principles calculations. (**f**) Normalized growth yield A as a function of *x* for Co (red) and CoO_x_ (blue) calculated by the theoretical equation (3). Solid and dashed line in (**e**) correspond to d = 0.757 nm and 0.692 nm, respectively. (**g**) Schematic illustration of the chirality shift from (6,5) to (6,4) achieved after pretreatment by considering the independent shift in d and *x*. (**h**) Experimental PL intensity ratio of (7,3)/(6,4) (red), (7,3)/(6,5) (black), and (6,4)/(6,5) (blue) as a function of H_2_/N_2_ flow ratio employed during the pretreatment process. Higher and lower ratio of H_2_/N_2_ result in stronger and weaker reduction of oxidized Co catalyst, resulting in pure and oxidized Co surface, respectively.

We then examined the growth process occurring after lifting off the cap structures. Recently, a promising growth model was reported by Artyukhov *et al*., revealing that the growth yield of SWNTs with a specific chirality can be explained by considering the nucleation probability and growth rate^29^.

1 $$N\propto exp(-\pi d(\gamma +{\gamma }^{^{\prime} }x)/{k}_{B}T)$$

2 $$R\propto \pi d\{exp(-\frac{2C}{{d}^{2}{k}_{B}T})\}\{x+exp(-\frac{E}{{k}_{B}T})\}$$

3 $$A=N\cdot R$$

where N, R, and A represent the nucleation probability, growth rate, and growth yield of SWNTs. The oxidation degree of Co catalyst directly influences the binding energy between the carbon atoms on the edge of SWNTs and catalyst surface (E_BE-M_), which is represented by γ in equation (1). γ′, variation of γ as a function of chiral angle calculated from the armchair side (*x*), should also be sensitive to the oxidation degree of the catalyst. C, *k*_*B*_, T, and E given in equation (2) correspond to the bending rigidity of graphene (3.9 eV/Å^2^)^30^, Boltzmann constant, synthesis temperature, and free-energy barriers for the initiation of a new atomic row on tube edge, respectively. To represent the catalyst achieved with and without pretreatment, pure Co (Co_55_) and oxidized Co (Co_33_O_22_) are considered, respectively (Fig. 5a). γ of (6,4) and (6,5) as well as γ′ of SWNTs grown on the surface of Co and oxidized Co are calculated by first-principles calculations (Fig. 5b–e). The binding energy obtained from first-principles calculations were divided by circumferential length of each tube (πd), giving γ for each chirality species. The d and *x* values for (6,4) and (6,5) SWNTs are: d_(6,4)_ = 6.92 Å, *x*_(6,4)_ = 6.59 deg. d_(6,5)_ = 7.57 Å, and *x*_(6,5)_ = 3 deg. In the near armchair region, γ′ can be regarded as a linear function of *x* ^29^. For oxidized Co, the values are as follows: γ_(6,4)_ = 6.82 eV/nm, γ_(6,5)_ = 6.34 eV/nm, and γ′ = 0.13 eV/nm deg. In contrast, for pure Co, the values are: γ_(6,4)_ = 7.26 eV/nm, γ_(6,5)_ = 7.1 eV/nm, and γ′ = 0.04 eV/nm deg. (Fig. 5e). With catalyst pretreatment, both γ_(6,4)_ and γ_(6,5)_ increase (Fig. 5e). Without pretreatment, γ strongly depends on *x*, resulting in a high γ′ value. On the other hand, the difference between γ_(6,4)_ and γ_(6,5)_ is low for the pretreated catalyst, resulting in a smaller γ′ value (Fig. 5e). The calculated values are substituted into the equation (3) to obtain the dependent of A as a function of *x*. We set the linear constant factor in equation (3) to be *x*_max_ of CoO_x_ = 3 (*x* of (6,5) SWNTs), because the dominant chirality species obtained without pretreatment (oxidized Co) is (6,5). The *x*_max_ corresponds to the value of *x* providing the maximum value for A. With catalyst pretreatment, *x*_max_ shifts to a higher degree of *x* up to ∼9.3 deg. (Fig. 5f). A similar calculation was also carried out for different d (d_(6,4)_ = 6.92 Å, d_(6,5)_ = 7.57 Å) and found that the shift of *x*_*max*_ for different d values is as small as 0.1 deg., denoting that shift of *x*_*max*_ is not induced by the difference in d but by the difference in γ′(Fig. 5f). As shown in Fig. 5f, *x*_max_ of (6,4) SWNTs ( = 6.59 deg.) is in between that of CoOx (3 deg.) and Co (∼9.3 deg). This is also in consistent with the experimental results demonstrating that the partial (not full) reduction (48%) of oxidized Co causes enhancement in the (6,4) SWNT growth (Fig. 3g and h).

The effect of pretreatment on the chirality selection can be summarized as follows. Initially, in the cap formation stage, E_BC-M_ increases with pretreatment, leading to a decrease in the diameter of SWNTs that can be lifted off from Co. After the lift off and during the axial growth, *x* dependence of E_BE-M_ (=γ) becomes weaker, resulting in the up shift of *x*_*max*_ for SWNTs having the highest growth yield (Fig. 5g). Overall, the significant transition of (6,5) to (6,4) SWNTs can be resulted by the shift in d toward smaller values and the up shift of *x* caused by the partial reduction of oxidized Co catalyst occurring during pretreatment (Fig. 5g).

If our model is reliable, the growth yield of other chirality species can also be changed by controlling the oxidation degree of Co catalyst. For example, with a further reduction of the oxidized Co surface, γ′ can be decreased further (we assume that γ′is linearly related to the oxidation degree of Co). In this case, *x*_*max*_ of SWNTs could be up shifted more than that of (6,4) SWNTs. (7,3) SWNTs exhibit a similar d (0.705 nm) as (6,4) SWNTs with a higher *x* value (13 deg.). Therefore, if further reduction can be achieved during pretreatment, the concentration of (7,3) SWNTs may increase. To verify this, we plotted the ratio of I_PL(7,3)_/I_PL(6,5)_ and I_PL(7,3)_/I_PL(6,4)_ as a function of H_2_/N_2_ ratio during pretreatment, which can tune the oxidation degree of Co. A high H_2_/N_2_ ratio indicates a lower degree of oxidation (close to pure Co). The concentration of (7,3) SWNTs increases with an increase in the H_2_/N_2_ ratio, indicating the reliability of our model (Fig. 5h). This shows that our concept of independently controlling d and *x* by tuning the oxidation degree of catalyst can be used for producing other chirality species.

## Conclusions

The preferential growth of (6,4) SWNTs is realized for the first time. Reduction of the catalyst occurs during the pretreatment process and the degree of oxidation of Co catalyst can be tuned with pretreatment carried out under the specific gas conditions. Systematic investigations and first-principles calculations show that on changing the oxidation degree on Co surface, E_BC-M_ and E_BE-M_ can be changed. The change in E_BC-M_ causes a downshift in the tube diameter due to the limitation of the lift off process. The chiral angle dependence of E_BE-M_ can be decreased by pretreatment, resulting in the shift of chiral angle *x* to a higher degree. By changing d and *x*, the dominant chirality species can be changed from (6,5) to (6,4) since the partial reduction of oxidized Co catalyst happens during pretreatment. This independent tuning of d and *x* by controlling the catalyst surface states can be a promising strategy for tuning the chirality of SWNTs, contributing toward realizing chirality selective growth of SWNTs for obtaining various chirality species.

## Methods

### Plasma CVD

A home-made plasma CVD system was used for diffusion plasma CVD. Before performing the plasma CVD growth, an electric furnace was heated to the desired temperature (typically around 600 °C). After reaching the desired temperature, an CH_4_ flow of 20 sccm (32 Pa) was employed. The catalyst holder was immediately transferred to the center area and subjected to rapid heating. As the catalyst holder was heated to the growth temperature, pressure was adjusted to 60 Pa. A radiofrequency power of 28 W (13.56 MHz) was supplied to the coils outside the quartz tube. A typical plasma irradiation time of 2 min was employed. After plasma CVD, when the catalyst holder temperature decreased to 500 °C, CH_4_ gas supply was stopped and the substrate was moved out of the electrical furnace.

### Catalyst preparation

Zeolite supported Co (CoMo) catalyst was prepared as follows. Initially, 0.5 wt% acetate tetrahydrate (and 0.5 wt% acetate dimer) was mixed with ferrierite zeolite (1 g). The prepared solid catalyst was then dissolved in 20 mL ethanol followed by performing ultrasonication for 15 min. After dispersing the catalyst, heating was performed under atmospheric conditions (80 °C, 24 h).

### Characterizations

The SWNT samples and catalysts were characterized using scanning electron microscopy (SEM; SU-70, Hitachi, Japan), TEM (JEM-2100F, JEOL, Japan), Raman scattering spectroscopy (HR-800, Horiba, Japan) using Ar laser excitation with a wavelength of 488 nm, AFM (JSPM-5400, JEOL, Japan), XPS (Ulvac-phi, ESCA1600, Japan), EDX (Oxford Aztec Energy X-Max, Oxford Instruments), and XAFS (EXAFS) (High Energy Accelerator Research Organization, Photon factory, Japan, measured by Toray Research Center, Inc.). The chirality of the SWNTs was evaluated with the help of PLE map (NanoLog, Horiba, Japan) and UV-Vis-NIR spectroscopy (V-7200HK, JASCO, Japan).

### First-principles calculations

Binding energy between graphene and Co crystal with and without oxygen atoms on the surface and that between nanotube edge and Co cluster were examined by the density functional theory (DFT) calculation using CASTEP^31^ in Materials Studio 2017^32^. The projector augmented wave (PAW) method^33^ was employed for the calculation of electronic states. The Perder-Burke-Ernzerhof (PBE) generalized gradient approximation (GGA)^34^ was employed for the exchange-correlation energy. The cutoff energy of 500 eV was employed. The k-point of 6 × 6 × 1 and the gamma point were employed for Brillouin zone sampling for interactions between the graphene and Co(0001) surface, and between the nanotube and the Co cluster, respectively. Three Co(0001) layers (each layer consists of four Co atoms) are placed in the slab cell of 4.91 × 4.91 × 18.00 Å. For the case of the Co surface with oxygen atoms, one or four oxygen atoms were placed on top of Co atom in second or third layer and prepared structures were optimized. Then, the graphene consisting of 8 atoms are placed on the Co surface at all commensurate orientations with the Co(0001) surface, that is, Rosei, top(fcc) and top(hcp) orientations^35^. Regarding the interaction between the nanotube edge and Co cluster, an optimized structure of (6,4) or (6,5) tube (two chiral units) is connected to on optimized structure of Co_55_ or Co_33_O_22_ cluster. The Co_33_O_22_ cluster is prepared by substituting random 22 positions of Co atoms with oxygen atoms and the structure was optimized. Merged structures of each combination were also optimized. The cubic cell of 20 Å^3^ was used for the optimization. The binding energy of each system was estimated by the energy difference between the energy of merged system and the sum of energies of each component.

### Data availability

The authors declare that the data supporting the findings of this study are available within the article and its supplementary data sets files.

## Electronic supplementary material

Supplementary Information
