# Supplementary material for: Preferential synthesis of (6,4) single-walled carbon nanotubes by controlling oxidation degree of Co catalyst
Source: Sci Rep. 2017 Sep 11;7:11149. doi: 10.1038/s41598-017-11712-0 (PMC5593930; doi:10.1038/s41598-017-11712-0)
Supplement: Supplementary file 1 — Supplementary Information [file 41598_2017_11712_MOESM1_ESM.doc]

Supplemental data set

Preferential synthesis of (6,4) single-walled carbon nanotubes by controlling oxidation degree of Co catalyst

Bin Xu^1^, Toshiro Kaneko^1^, Yasushi Shibuta^2^, and Toshiaki Kato^1^*

1 Department of Electronic Engineering, Tohoku University, Aoba 6-6-05, Aramaki, Aoba-ku, Sendai 980-8579, Japan

2 Department of Materials Engineering, The University of Tokyo, 7-3-1, Hongo, Bunkyo-ku, Tokyo 113-8656, Japan

Table S1. Summary of various methods for chirality selective synthesis of SWNTs.


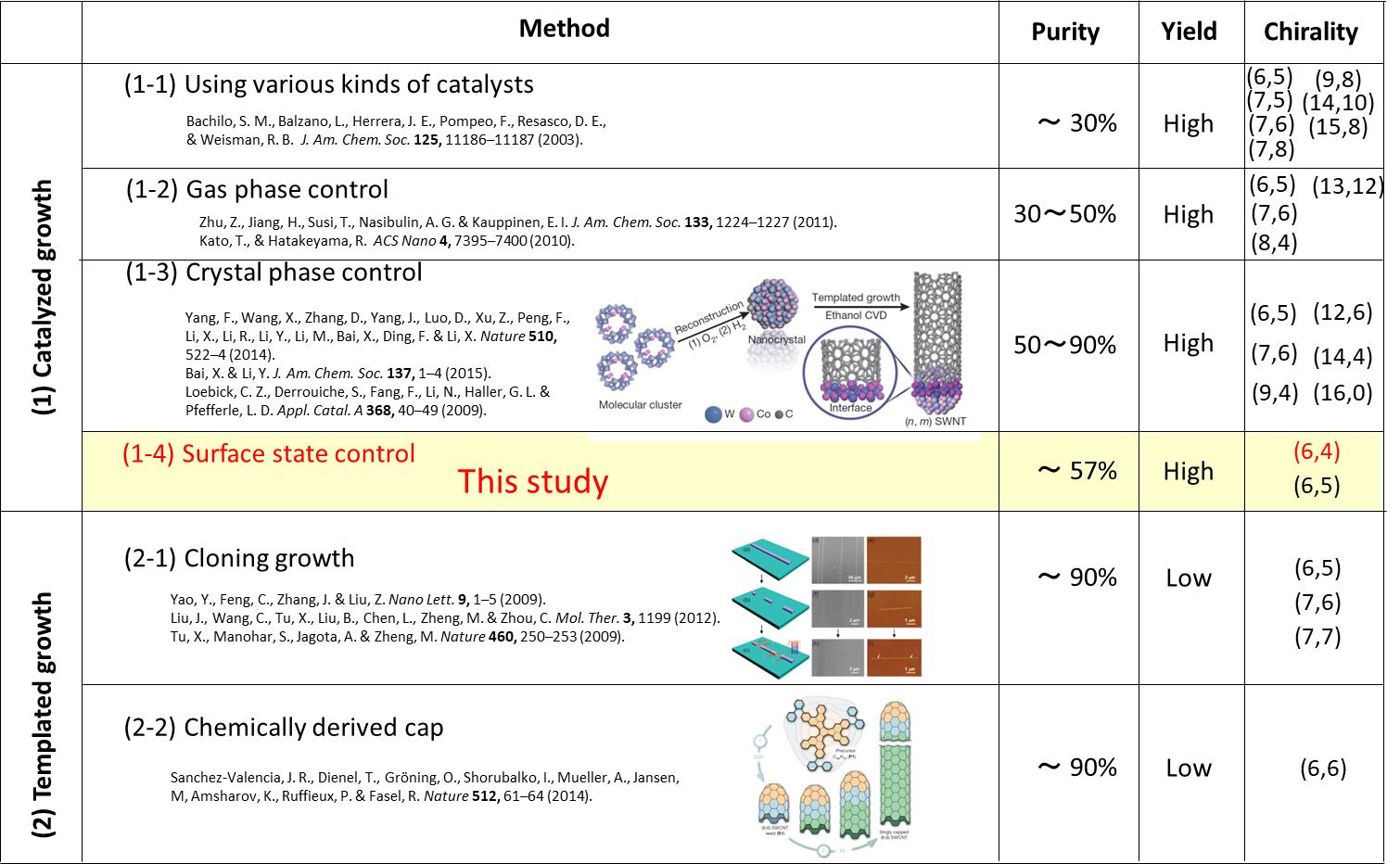


Fig. S1. Typical mass spectra of gas phase during the pretreatment.


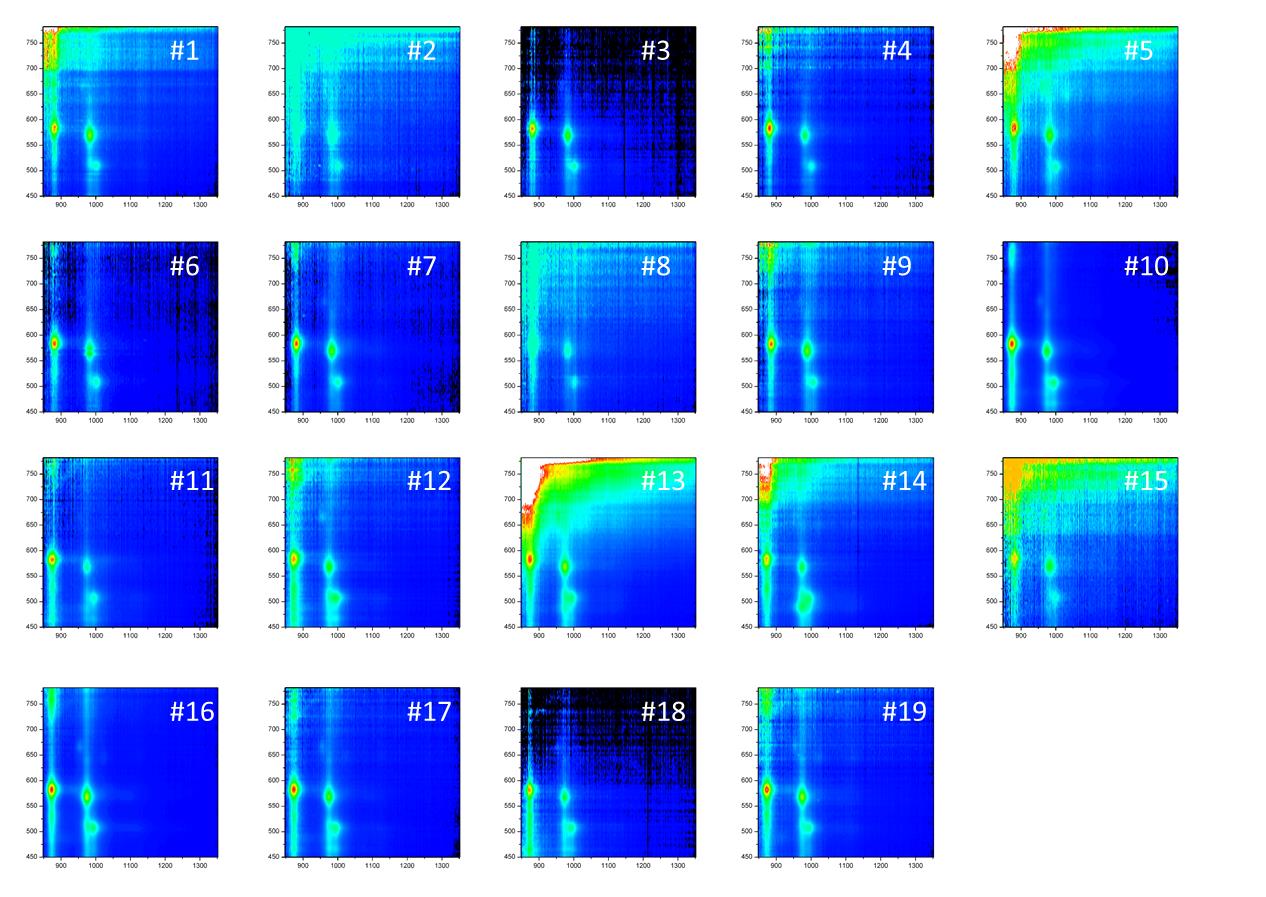


Fig. S2. PLE maps of SWNTs grown under the same condition (best condition of (6,4) rich growth) with different experiments (#1-#19).


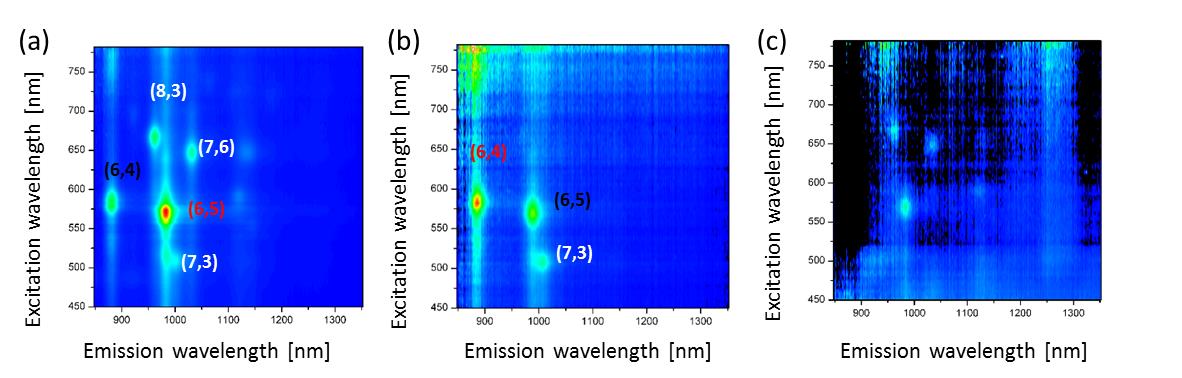


Fig. S3. PLE maps of SWNTs synthesized using (a) Co without pretreatment, (b) Co with pretreatment, (c) Mo without pretreatment.


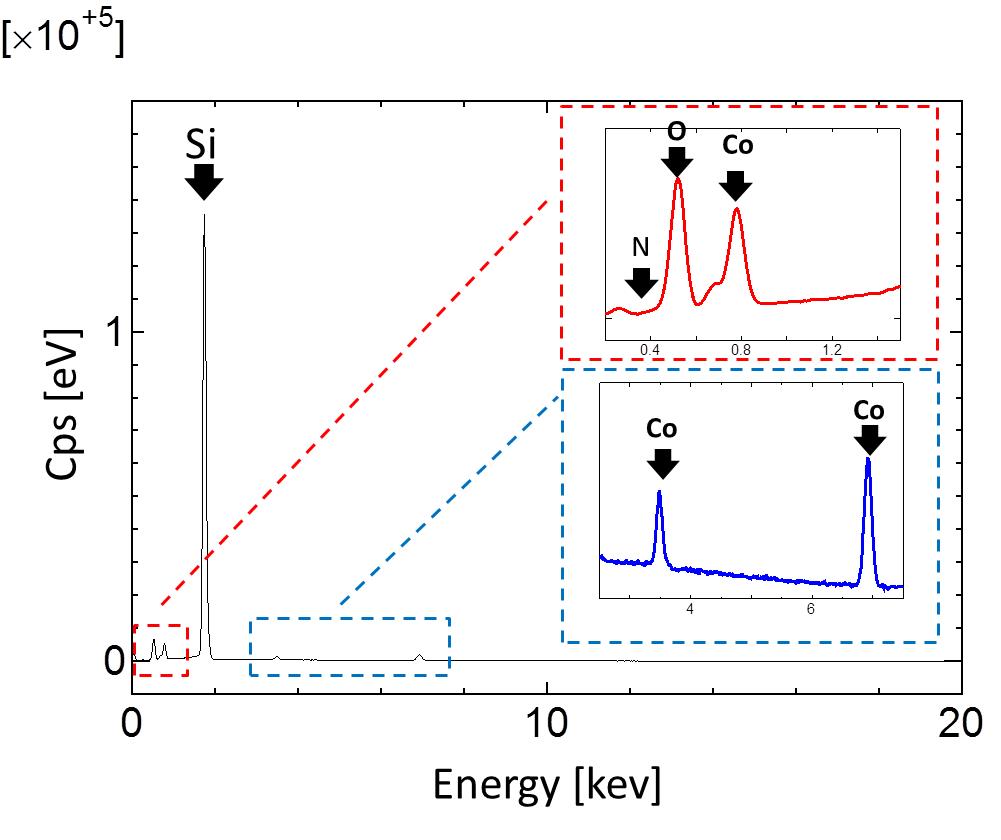


Fig. S4. Typical EDX spectra of Co film after pretreatment. Inset shows the magnified spectra of O and Co region.


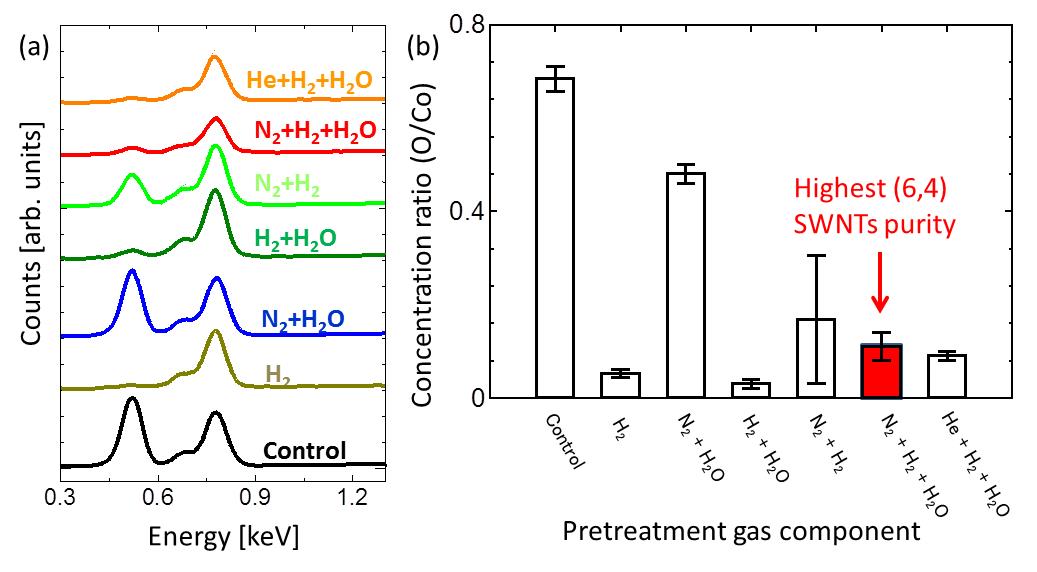


Fig. S5. Typical (a) EDX spectra and (b) bar graph of concentration ratio of O to Co of Co film treated by various gas atmosphere.


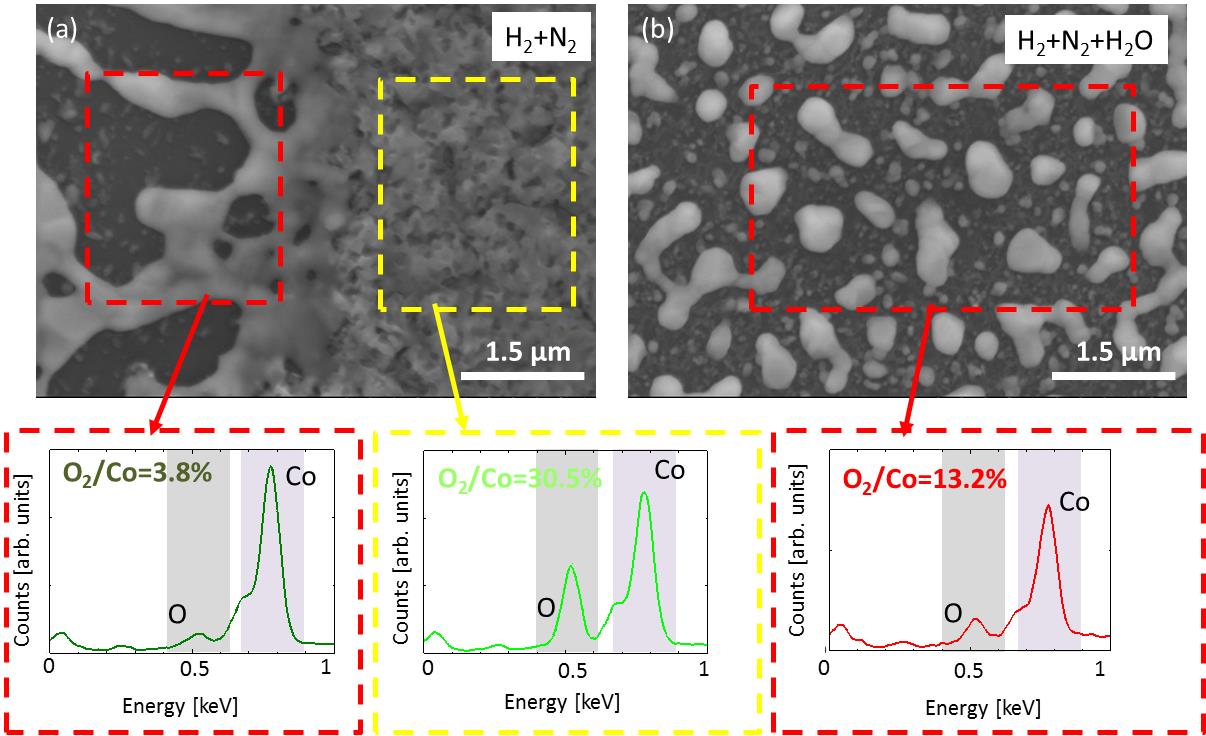


Fig. S6. Typical SEM images and EDX spectra of Co film pretreated under (a) H_2_+N_2_ and (b) H_2_+N_2_+H_2_O atmosphere.


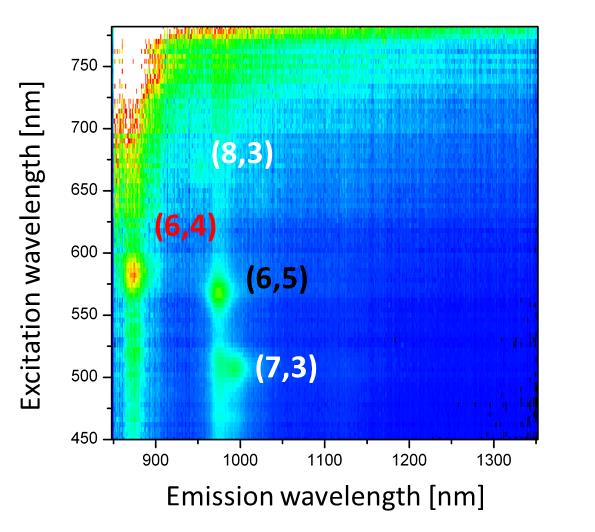


Fig. S7. PLE map of SWNTs grown with Co catalyst pretreated by H_2_+H_2_O+He atmosphere.


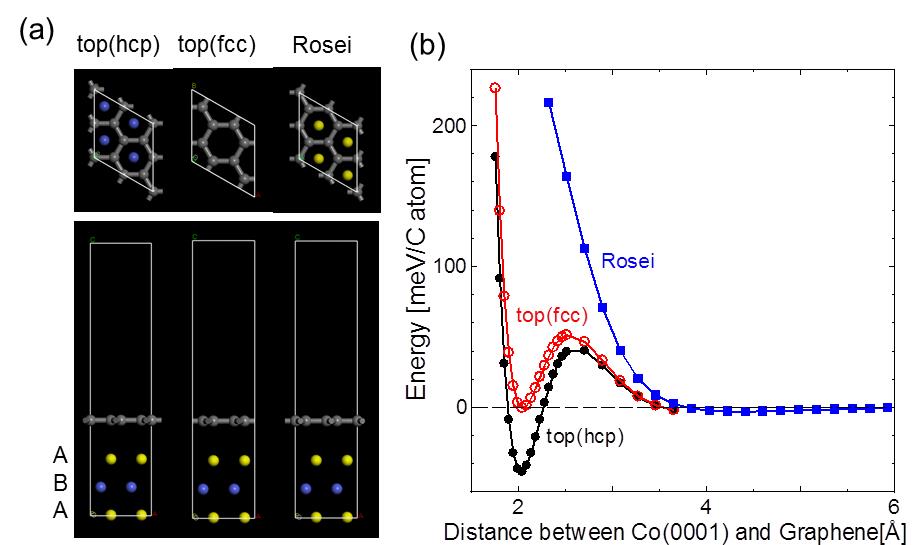


Fig. S8. (a) Three Atomistic configuration of three commensurate orientations between graphene and Co(0001) surface: (top(hcp), top(fcc) and Rosei orientations from the left). Blue and yellow spheres represent cobalt atoms in A and B layers, respectively, and gray ones represent carbon atoms. (b) Binding energy per carbon atom between Co(0001) surface and graphene as a function of interlayer distance.


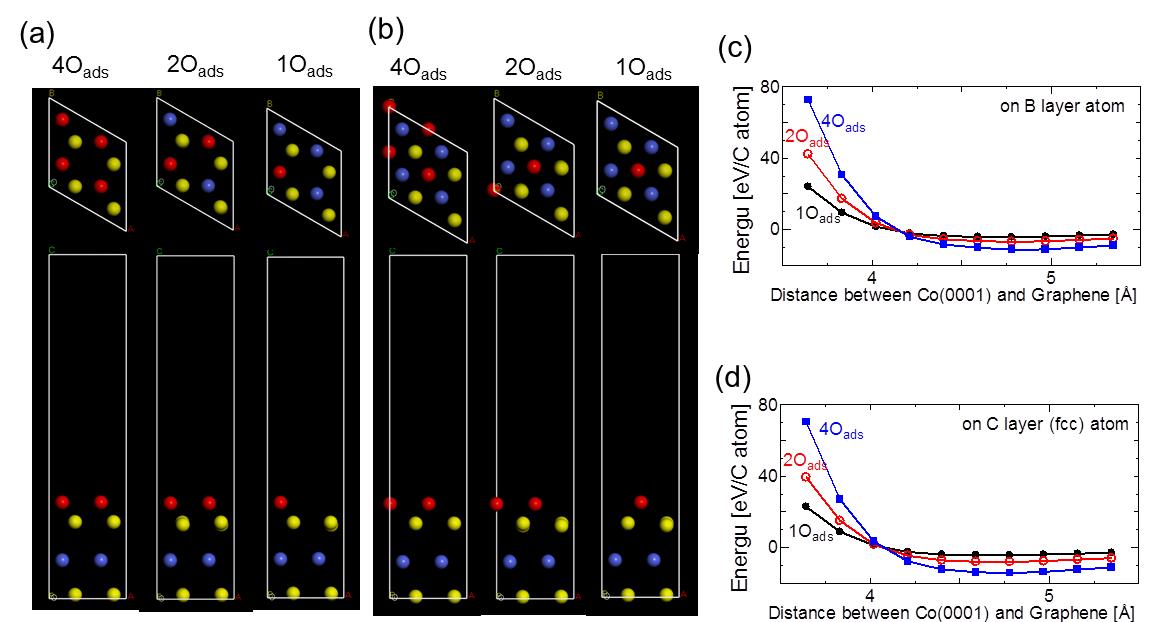


Fig. S9. First principle calculation of the interaction between graphene and Co lattice with oxygen atoms on the surface. (a-b) Optimized structures of Co(0001) layer with one, two and four oxygen atoms on (a) top(hcp) and (b) top(hcp) sites of the surface. (c-d) Binding energy per carbon atom between graphene and Co surface with oxygen atoms on (a) top(hcp) and (b) top(hcp) sites of the surface a function of interlayer distance.
